# Hereditary Drunkenness

**Published:** 1915-10

**Authors:** 


**Hereditary Drunkenness.** Geo. B. Simpson, of Parkersbury, W. Va., Med. Summary, Sept., relates a case in which a habitually drunken father impregnated his wife while drunk. The offspring, now a young man, presents the constant appearance of drunkenness except after taking a drink, when he becomes sober.

